# Association between *MBL2* haplotypes and dengue severity in children from Rio de Janeiro, Brazil

**DOI:** 10.1590/0074-02760190004

**Published:** 2019-05-23

**Authors:** Alice Maria de Magalhães Ornelas, Caroline Xavier-de-Carvalho, Lucia Elena Alvarado-Arnez, Marcelo Ribeiro-Alves, Átila Duque Rossi, Amilcar Tanuri, Renato Santana de Aguiar, Milton Ozório Moraes, Cynthia Chester Cardoso

**Affiliations:** 1 Universidade Federal do Rio de Janeiro, Instituto de Biologia, Departamento de Genética, Laboratório de Virologia Molecular, Rio de Janeiro, RJ, Brasil; 2 Fundação Oswaldo Cruz-Fiocruz, Instituto Oswaldo Cruz, Laboratório de Hanseníase, Rio de Janeiro, RJ, Brasil; 3 Fundação Oswaldo Cruz-Fiocruz, Instituto Nacional de Infectologia Evandro Chagas, Laboratório de Pesquisa Clínica em DST-AIDS, Rio de Janeiro, RJ, Brasil

**Keywords:** dengue, polymorphisms, MBL2, CCR5, ITGB3, CLEC5A

## Abstract

**BACKGROUND:**

Dengue is an arthropod-borne viral disease with a majority of asymptomatic individuals and clinical manifestations varying from mild fever to severe and potentially lethal forms. An increasing number of genetic studies have outlined the association between host genetic variations and dengue severity. Genes associated to viral recognition and entry, as well as those encoding mediators of the immune response against infection are strong candidates for association studies.

**OBJECTIVES:**

The aim of this study was to investigate the association between *MBL2*, *CLEC5A*, *ITGB3* and *CCR5* genes and dengue severity in children.

**METHODS:**

A matched case-control study was conducted and 19 single nucleotide polymorphisms (SNPs) were investigated.

**FINDINGS:**

No associations were observed in single SNP analysis. However, when *MBL2* SNPs were combined in haplotypes, the allele rs7095891G/rs1800450C/ rs1800451C/rs4935047A/rs930509G/rs2120131G/rs2099902C was significantly associated to risk of severe dengue under α = 0.05 (aOR = 4.02; p = 0.02). A second haplotype carrying rs4935047G and rs7095891G alleles was also associated to risk (aOR = 1.91; p = 0.04).

**MAIN CONCLUSIONS:**

This is the first study to demonstrate the association between *MBL2* haplotypes and dengue severity in Brazilians including adjustment for genetic ancestry. These results reinforce the role of mannose binding lectin in immune response to DENV.

Dengue is a neglected tropical disease caused by Dengue virus (DENV), which has four circulating serotypes (DENV-1 to 4) and is transmitted through the bite of *Aedes* mosquitoes. In humans, the majority of infections are asymptomatic or presenting mild flu-like symptoms, known as dengue fever. However, a small proportion of the cases may also present the life-threatening forms such as dengue shock syndrome.^1^

Recent estimates suggest an annual occurrence of 390 million infections worldwide.^2^ Dengue infection affects infants, young children and adults, and is responsible for 250,000-500,000 hospitalisations and approximately 25,000 deaths each year, mostly among children.^3^ In Americas, The Southern Cone subregion reported a total of 254,453 dengue cases in 2017 from which, 99% were reported in Brazil.^4^

Several risk factors influence evolution to severe disease in dengue-infected individuals. Among these factors are secondary infection, known as antibody-dependent enhancement, viral strain differences, viral load, potent host inflammatory immune response, that includes T-cell response, cytokine storm, vascular leakage and complement activation and host genetic background.^1^ ^,^ ^5^ Therefore, disease severity is a complex phenomenon and depends on numerous interactions between human genetic and immunological characteristics as well as viral factors.^1^ ^,^ ^6^ ^,^ ^7^

The influence of the host genetic background in susceptibility to severe dengue has been evidenced for different populations using either candidate genes or genome-wide approaches.^6^ A genome-wide association study has reported the association between *MICB* and *PLCE* genes and dengue shock syndrome in children from Vietnam.^8^ Moreover, dengue outcomes have already been associated to HLA alleles as well as genes encoding the cell receptor DC-SIGN, *CLEC5A*, Fc receptors, and molecules such as CTLA-4, TGF-β and MBL-2.^6^ ^,^ ^7^ However, conflicting results are still observed and the complete dissection of host genetic factors influencing dengue clinical course still depends on the development of independent studies including a careful control for genetic background.

Mannose-binding lectin (MBL) is a pattern recognition receptor which recognises specific sugar groups on the surface of various microorganisms and triggers complement activation to promote pathogen elimination.^9^ In dengue infection, this lectin is required for complement-mediated viral neutralisation.^10^ MBL is encoded by *MBL2* gene, which comprises four exons. Variations at codons 54 (rs1800450), 57 (rs1800451) and 52 (rs5030737) as well as promoter polymorphisms have been associated to MBL levels and activity.^11^ ^,^ ^12^ However, the association between *MBL2* gene and dengue is still controversial and remains to be clearly characterised.

Another common candidate for association studies in dengue is the C-type lectin domain family 5, member A (*CLEC5A*) which has been implicated in dengue lethal outcomes.^13^ Upon infection, *CLEC5A* directly interacts with DENV and stimulates the release of proinflammatory cytokines.^13^ We have recently demonstrated an association between the polymorphism rs1285933, located at *CLEC5A* 3’UTR region, and severe dengue in two Brazilian cohorts.^14^ ^,^ ^15^

In addition to the classical candidates, other genes and molecules have also been investigated in dengue infection, including the coreceptor *CCR5* and integrins. Recently, *CCR5* has been described as a host factor required not only for DENV-2 replication but also for the development of the disease in macrophages.^16^ β3 integrins are abundant on the surface of vascular endothelial cells and platelets, and play an important role in maintaining vascular integrity,^17^ suggesting a possible association to severe dengue. In fact, α5β3 integrins are commonly used as receptors or coreceptors by several viruses, including DENV.^18^

We have previously investigated the role of 11 candidate single nucleotide polymorphisms (SNPs) in four genes including *CLEC5A*, which was associated to dengue severity, and *CCR5*.^14^ In the present study, we have increased the number of genes/polymorphisms investigated in the same cohort by including new markers at *CLEC5A* and *CCR5*, in addition to 13 markers at *MBL2* and *ITGB3* genes. Proportions of African, European and Native American genetic ancestries were also estimated to adjust for confounding.

## MATERIALS AND METHODS

*Subjects and study design* - The case group included 87 children with severe dengue admitted in four pediatric intensive care units from Rio de Janeiro - IFF/FIOCRUZ; Instituto de Puericultura e Pediatria Martagão Gesteira, IPPMG/UFRJ; Prontobaby, Hospital da Criança; and Hospital Municipal Jesus - between 2007 and 2008, aged up to 18 years. Severe dengue was defined in a previous study, according to clinical symptoms as described by Brazilian Ministry of Health and WHO.^14^ ^,^ ^19^ The patients exhibited symptoms consistent with shock syndrome, including slow capillary filling, cold clammy skin, filiform or absent pulse in the presence of either hypotension for age or narrow pulse pressure. Other typical symptoms of dengue were considered such as fever, hemorrhagic manifestations, hemoconcentration, constant vomits, persistent abdominal pain, hypotension, pleural or pericardial effusion or ascites and high levels of AST and ALT. Detailed information about case definition, inclusion/exclusion criteria and ethics statement was described in a previous study.^14^ A control group was selected among neighbors and cases’ household members matched by age (up to three years older or younger than the cases). A total of 325 controls were initially included in the study. After serological tests, the rate of dengue IgG seropositivity was 60.6% and, therefore, the final control group included 197 individuals. Considering a minor allele frequency of 0.05, α = 0.05 and the number of cases and IgG+ controls available, the minimum OR value to achieve 80% of power was 3.4.

Individual estimates of African, European, and Native American genetic ancestries were determined using 28 ancestry informative markers, as described.^20^ Distributions of age, gender and genetic ancestry are shown for cases and IgG+ controls in Table I.

**TABLE I:** Distribution of age, gender and genetic ancestry in dengue severe cases and controls

Variable	Cases N = 87	IgG+ controls N = 197
Age (years)	10.03 ± 3.79	10.02 ± 3.57
Gender		
Male	42 (48%)	111 (44%)
Female	45 (52%)	86 (56%)
Genetic ancestry (%)		
European^*^	58 ± 20	50 ± 24
African^*^	27 ± 18	35 ± 22
Native American	15 ± 16	16 ± 18

Results are represented as mean standard ± deviation for age and genetic ancestry. *: p < 0.05 for comparisons between cases and IgG+ controls.

*Candidate SNPs selection* - Candidate SNPs were selected for genotyping based on their classification as a tag to a haplotype or location in coding and/or regulatory regions of genes using HapMap database (www.hapmap.org), Snpper tool (http://snpper.chip.org/) and/or dbSNP database (http://ncbi. nlm.nih.gov/SNP/). SNPs with minor allele frequencies (MAF) of at least 0.05 at *MBL2*, *ITGB3*, *CCR5* and *CLEC5A* genes were selected. For *CLEC5A*, a 10 kb upstream region was also analysed for SNP selection to increase coverage. The tag SNPs were selected from HapMap database (phase II Oct 14, on NCBI B36 assembly, dbSNP b126) using data from YRI (Yoruba in Ibadan, Nigeria) and CEU (Utah residents with ancestry from northern and western Europe) populations. A minor allele frequency of 0.01 and r2 ≥ 0.8 was applied as criteria for tag definition. According to these criteria, 22 polymorphisms were selected for analysis [Supplementary data (Table I)], including eight polymorphisms at *MBL2* (rs930509, rs4935047, rs2099902, rs2120131, rs1800451, rs1800450, rs5030737 and rs7095891), four SNPs at *CLEC5A* (rs1285950, rs1594777, rs1285948 and rs2570407), six at *ITGB3* (rs11655943, rs4629025, rs884696, rs7209700, rs3809865 and rs5918) and, finally, four SNPs at *CCR5* gene (rs1800940, rs2856762, rs3176763 and rs3087253).

*SNP genotyping* - Genomic DNA was extracted from peripheral blood by using a salting-out protocol. All SNPs were genotyped using 5’ nuclease assays for allelic discrimination by real-time polymerase chain reaction (PCR) using QuantStudio™ 12K Flex Real-Time PCR System with OpenArray® Block (ThermoFisher Scientific, MA, USA). Reactions were performed in customised arrays according to the manufacturer’s instructions. The SNPs rs1285950, rs1594777, and rs1285948 at *CLEC5*, could not be included in the array due to a failure in probe design.

*In silico analysis of MBL2 SNPs* - Potential functional impact for *MBL2* SNPs was assessed using five different algorithms. Combined annotation dependent depletion (CADD) (v1.4) and deleterious annotation of genetic variants using neural networks (DANN) are evolutionary-based approaches which infer functional impact through data simulation. Functional analysis through hidden markov models (FATHMM-MKL) is an integrative algorithm that uses functional annotations and sequence conservation data through machine learning approach. Finally, PolyPhen2 and sorting intolerant from tolerant (SIFT) are structure-based approaches that infer functional impact according to sequence conservation and severity of the amino acid change.^21^ Functional impact scores were interpreted here following developers’ recommendations. In addition, data from The Genotype-Tissue Expression (GTEx) project^22^ were also analysed to search for eQTLs using each SNP as query in the GTEx portal.

*Statistical analysis* - Deviations from Hardy-Weinberg equilibrium (HWE) were assessed by Chi-square test using samples from IgG+ controls (N = 197). Frequencies of each genotype, allele, and minor allele carriers in cases and controls were determined by direct counting. The genotypic, allelic and minor allele carriers association with severe dengue were assessed using odds-ratios (OR) and corresponding 95% confidence interval (95%CI) estimated by the fit of logistic generalised linear mixed effect models (L-GLMM) by maximum likelihood, where clusters of neighbors and/or household members were modeled as aleatory effects. Degrees of freedom for fitted effects were approximated by the Satterthwaite method.^23^ Gender and African and Native American ancestry estimates were included as fixed effects in multivariate analysis to adjust for confounding (aOR). Linkage disequilibrium (LD) patterns were assessed using the r2 statistic also using samples from the complete control group. Haplotype L-GLMM analysis was performed with SNPs, not in LD. The allelic phases were estimated by the expectation-maximisation method, and phase uncertainties were treated as sample weights in model fittings. The same strategy was applied to determine the frequency of each haplotype among Africans (AFR), Europeans (EUR), Mixed americans (AMR) and East and South Asians (EAS and SAS) populations from 1000 Genomes Phase 3.^24^ Allelic, genotypic, minor allele carriers, and Haplotypic L-GLMM were adjusted for multiple comparisons by false discovery rate (FDR). All analysis was performed in R version 3.4.4.

*Ethics statement* - This study was approved by research ethics committee (CAAE3723.0.000.009-08 Instituto Nacional de Infectologia Evandro Chagas/FIOCRUZ). All procedures were performed in accordance with the guidelines of the Helsinki Declaration, as previously described.^14^ Informed consent was obtained in two copies from the parents of all subjects enrolled in the study. A signed copy saved with the clinical researcher and the other with the family.

## RESULTS

*Quality control and single SNP association analyses* - A total of 19 candidate SNPs [Supplementary data (Table I)] was genotyped in 87 cases and 325 controls. SNPs rs5030737 (*MBL2*) and rs1800940 (*CCR5*) were excluded from analysis because they were not informative (monomorphic) in our sample. The remaining 17 SNPs were successfully genotyped, with call rates of at least 90%. In all SNPs tested, genotype frequencies were found in HWE. Results of linkage disequilibrium analyses showed moderate LD between SNPs rs2099902 and rs2120131 at *MBL2* (r^2^ = 0.68) and also between rs4629025 and rs7209700 at *ITGB3* (r^2^ = 0.61). A minimum r^2^ of 0.8 was used as cutoff for *tag* SNPs definition. Therefore, all SNPs were included in association analyses.

Frequencies of each SNP were compared between severe cases and IgG+ controls. Results of L-GLMM did not show any statistically significant association when each SNP was analysed independently either before or after adjustment for gender and proportions of African and Native American ancestries [Table II; Supplementary data (Table II)].

**TABLE II:** Distribution of *MBL2* single nucleotide polymorphisms (SNPs) and analysis of association to dengue severity in children from Rio de Janeiro

SNP	Genotypes	Cases^*^	Controls^*^	OR (95% CI; p-value)	OR (95% CI; p-value)^**^
rs7095891	GG	42 (50.0)	73 (39.7)	reference	Reference
	GA	36 (42.9)	82 (44.5)	0.78 (0.44 - 1.41; p = 0.42)	1.01 (0.51 - 2.00; p = 0.98)
	AA	6 (7.1)	29 (15.8)	0.44 (0.16 - 1.16; p = 0.10)	0.42 (0.13 - 1.34; p = 0.14)
	Total	84	184		
	A carriers	42 (50.0)	111 (60.3)	0.70 (0.40 - 1.23; p = 0.21)	0.85 (0.44 - 1.64; p = 0.63)
rs1800450	CC	65 (77.4)	145 (81.0)	Reference	Reference
	CT	19 (22.6)	31 (17.3)	1.43 (0.75 - 2.73; p = 0.28)	1.47 (0.73 - 2.99; p = 0.28)
	TT	0 (0)	3 (1.7)	n.d	n.d
		84	179		
	T carriers	19 (22.6)	34 (19.0)	1.31 (0.69 - 2.48; p = 0.41)	1.32 (0.66 - 2.63; p = 0.43)
rs1800451	CC	68 (85.0)	145 (83.8)	Reference	Reference
	CT	12 (15.0)	27 (15.6)	1.26 (0.57 - 2.78; p =0.57)	1.51 (0.63 - 3.64; p = 0.35)
	TT	0 (0.0)	1 (0.6)	n.d	n.d
	Total	80	173		
	T carriers	12 (15.0)	28 (16.2)	1.15 (0.53 - 2.53; p = 0.72)	1.33 (0.56 - 3.13; p = 0.52)
rs4935047	AA	21 (29.2)	69 (40.6)	Reference	Reference
	AG	34 (47.2)	74 (43.5)	1.18 (0.57 - 2.44; p = 0.65)	1.17 (0.53 - 2.56; p = 0.70)
	GG	17 (23.6)	27 (15.9)	2.02 (0.86 - 4.75; p = 0.11)	1.90 (0.69 - 5.24; p = 0.21)
	Total	72	170		
	G carriers	51 (70.8)	101 (59.4)	1.42 (0.73 - 2.75; p = 0.3)	1.34 (0.64 - 2.79; p = 0.44
rs930509	GG	55 (67.9)	135 (75.4)	Reference	Reference
	GC	25 (30.9)	42 (23.5)	1.36 (0.68 - 2.68; p = 0.38)	1.17 (0.55 - 2.52; p = 0.68)
	CC	1 (1.2)	2 (1.1)	n.d	n.d
	Total	81	179		
	C carriers	26 (32.1)	44 (24.6)	1.32 (0.67 - 2.61; p = 0.43)	1.13 (0.52 - 2.43; p = 0.76)
rs2120131	TT	35 (45.5)	91 (54.2)	Reference	Reference
	TG	36 (46.7)	62 (36.9)	1.79 (0.91 - 3.51; p = 0.09)	1.53 (0.77 - 3.05; p = 0.23)
	GG	6 (7.8)	15 (8.9)	1.43 (0.42 - 4.91; p = 0.57)	1.20 (0.33 - 4.36; p = 0.78)
	Total	77	168		
	G carriers	42 (54.5)	77 (45.8)	1.73 (0.90 - 3.32; p = 0.10)	1.48 (0.76 - 2.89; p = 0.25)
rs2099902	TT	33 (42.3)	70 (40.2)	Reference	Reference
	TC	34 (43.6)	77 (44.3)	1.01 (0.54 - 1.89; p = 0.97)	1.28 (0.65 - 2.56; p = 0.47)
	CC	11 (14.1)	27 (15.5)	0.97 (0.37 - 2.49; p = 0.94)	1.15 (0.41 - 3.25; p = 0.79)
	Total	78	174		
	C carriers	45 (57.7)	104 (59.8)	1.04 (0.44 - 2.49; p = 0.92)	1.00 (0.39 - 2.61; p = 0.99)

*: results are shown as N (%) for cases and IgG+ controls. **: odds-ratios (OR) and p-value adjusted for gender and African and Native American genetic ancestries; n.d = not done.

*Haplotype analyses* - When *MBL2* SNPs were combined in haplotypes, two combinations carrying allele rs7095891G (haplotypes 2 and 6), in addition to other regulatory variations, were associated to increased risk of severe dengue (Table III). Haplotype 2 (rs7095891G/ rs1800450C/ rs1800451C/ rs4935047A/ rs930509G/ rs2120131G/ rs2099902C) was significantly associated to risk (aOR = 4.02; 95%CI: 1.23-13.09; p = 0.02) as compared to the reference (A/C/C/A/G/T/T). Haplotype 6 (G/C/C/G/G/T/T), with higher frequency, and carrying rs4935047G in addition to rs7095891G was also associated to risk (aOR = 1.91; 95%CI: 1.02-3.6; p = 0.04). After FDR adjustment, however, all p-values were above 0.05, due to a clear loss in statistical power.

**TABLE III:** Association between *MBL2* haplotypes and dengue severity in children from Rio de Janeiro

Haplotype	rs7095891/ rs1800450/ rs1800451/ rs4935047/ rs930509/ rs2120131/ rs2099902	Controls^*^	Cases^*^	Odds-ratios (OR) (95%CI; p)	OR (95%CI; p)^**^
1	A/C/C/A/G/T/T	86 (29)	26 (19)	Reference	Reference
2	G/C/C/A/G/G/C	6 (2)	8 (6)	4.32 (1.36-13.8; p = 0.01)	4.02 (1.23-13.09; p = 0.02)
3	G/C/C/G/G/G/C	19 (6)	9 (6)	1.52 (0.6-3.88; p = 0.37)	1.75 (0.66-4.68; p = 0.26)
4	G/C/C/A/G/T/C	19 (6)	4 (3)	0.71 (0.22-2.29; p = 0.57)	1.01 (0.3-3.4; p = 0.99)
5	G/C/C/A/C/T/T	34 (11)	19 (14)	1.89 (0.92-3.86; p = 0.08)	1.69 (0.79-3.6; p = 0.17)
6	G/C/C/G/G/T/T	68 (23)	39 (28)	1.96 (1.08-3.54; p = 0.03)	1.91 (1.02-3.6; p = 0.04)
7	A/C/T/A/G/G/C	26 (9)	11 (8)	1.4 (0.6-3.25; p = 0.43)	1.64 (0.69-3.91; p = 0.26)
8	G/T/C/G/G/G/C	22 (7)	12 (9)	1.83 (0.79-4.23; p = 0.16)	1.69 (0.7-4.07; p = 0.24)

*: haplotype frequencies were estimated by maximum likelihood and represented as N (%). Haplotypes with frequencies bellow 0.02 were excluded from analysis. **: results were adjusted for gender and African and Native American genetic ancestries. Statistically significant associations (α = 0.05) are shown in bold. Alleles that differ from reference were underlined in the associated haplotypes.

Comparisons with data obtained from populations of 1000 Genomes project showed similar frequencies of the high risk haplotype 2 between data from the present study (0.03, including cases and controls) and among Africans (AFR; 0.04), Mixed Americans (AMR; 0.02) and Europeans (EUR; 0.04). The lowest frequencies were observed among East and South Asians (0.003 and 0.007, respectively). For haplotype 6, frequencies varied from 0.12 (AFR) to 0.40 (AMR) [Supplementary data (Table III)].

Considering the moderate LD between SNPs rs2099902 and rs2120131 (r^2^ = 0.68), haplotype analyses were also performed removing one of these markers (rs2099902). Results obtained showed that the haplotype rs7095891G/ rs1800450C/ rs1800451C/ rs4935047G/ rs930509G/ rs2120131T was associated to risk, with OR values similar to those obtained in the complete haplotype 6 (aOR = 1.84; 95%CI: 0.99-3.44; p = 0.055), using A/C/C/A/G/T as reference. On the other hand, the association between the low frequency haplotype G/C/C/A/G/G including rs7095891G and rs2120131G alleles, was softened after removing rs2099902 (aOR = 2.69 *vs* 4.02), with a consequent reduction in power (p = 0.1). SNPs at *ITGB3* and *CCR5* genes were also combined in haplotypes, but no association was found (data not shown).

*In silico prediction of MBL2 SNPs functional effect* - Possible functional impacts of *MBL2* variations were investigated *in silico*. Results obtained from five different algorithms have classified the nonsynonymous polymorphisms rs1800450, rs1800451 and rs5030737 as functional or potentially pathogenic (Table IV). Moreover, when the intron variations rs930509 and rs4935047 were queried at GTEx browser, allele G at both sites was associated to higher *MBL2* expression in liver (p = 7.2 e^-13^ for rs930509 and p = 1.3 e^-4^ for rs4935047).

**TABLE IV:** *In silico* prediction of functional effects of *MBL2* single nucleotide polymorphisms (SNPs) according to five algorithms

SNP ID	Gene region (function)	CADD (PhRed score)	DANN score	FATHMM (MKL score)	PolyPhen-2	SIFT
rs2099902	3’UTR	0.520	n.d	0.00014	n.d	n.d
rs2120131	3’UTR	1.190	n.d	0.03244	n.d	n.d
rs930509	Intron	3.006	n.d	0.06633	n.d	n.d
rs4935047	Intron	6.217	n.d	0.02010	n.d	n.d
rs1800451	Exon	24.6	0.997	0.675	Damaging	0.001
rs1800450	Exon	25.5	0.999	0.691	Damaging	0.03
rs5030737	Exon	25.8	0.999	0.091	Damaging	0.001
rs7095891	Upstream	0.094	n.d	0.00396	n.d	n.d

Scores higher than 20 (combined annotation dependent depletion ― CADD), 0.91 (deleterious annotation of genetic variants using neural networks ― DANN), 0.5 (functional analysis through hidden markov models ― FATHMM-MKL) and lower than 0.05 (sorting intolerant from rolerant ― SIFT), were considered pathogenic; n.d: not done.

## DISCUSSION

Dengue susceptibility is a complex phenotype that depends on components intrinsic to the virus and the host. Genetic heterogeneity of molecules involved in the host immune response to DENV infection has been supported to play a key role in the development of severe disease.^6^ Here, we investigated the association between severe dengue and 19 candidate SNPs in genes encoding critical molecules involved in the host immunological resistance against DENV infection, including mannose-binding lectin, *CLEC5A*, β3 integrin, and *CCR5*.

Mannose-binding lectin acts in first line response to DENV.^6^ ^,^ ^10^ A study of Vietnamese subjects has detected no association between the variation Gly54Asp (rs1800451) and dengue clinical outcomes.^25^ By contrast, studies from Northeast Brazilian groups have both reported associations between combinations of functional *MBL2* exon 1 SNPs at codons 52 (rs5030737), 54 (rs1800450) and 57 (rs1800451) and dengue outcomes such as thrombocytopenia^26^ and also dengue haemorrhagic fever (DHF).^27^ Here, we have detected an association between two *MBL2* haplotypes and increased risk of severe dengue. The strongest association was observed for haplotype 2, which differed from the reference haplotype by carrying the alleles rs7095891G, rs2099902C and rs2120131G (aOR = 4.02; p = 0.02). As expected, similar frequencies of this haplotype were obtained among AFR, AMR and EUR populations from 1000 Genomes project (0.04, 0.02 and 0.04, respectively). The lowest frequencies were observed among East (0.003) and South Asians (0.007) [Supplementary data (Table III)], suggesting that large sample sizes would be required to replicate our findings in populations with this genetic background.

The SNP rs7095891 is located at *MBL2* 5’UTR, while rs2099902 and rs2120131 are located at 3’UTR region. In silico predictions have suggested that 3′ UTR SNPs such as rs2099902 and rs2120131, could affect miRNA binding site, while intronic and 5′ near gene SNPs as rs7095891 could affect transcription binding sites.^28^ In fact, data obtained from the GTEx project have suggested that the intron variations rs930509 and rs4935047 act as eQTLs for *MBL2* gene in liver. However, data from other tissues and cell types were not available for analysis. The allele rs7095891G also referred as MBL2*Q, has also been associated to variations in *MBL2* serum levels depending on combinations with promoter SNPs.^11^ ^,^ ^12^ Taken together, the literature data suggest that *MBL* levels may be regulated by variations along all gene sequence.

The OR values towards risk suggest that haplotypes 2 and 6 might be associated to lower *MBL* levels, since deficiency of this receptor have been associated to severe dengue phenotypes.^27^ ^,^ ^29^ However, further analyses are still required to determine the correlation between the associated haplotypes and MBL-2 levels during DENV infection. A possible interaction between genetic variations and classical risk factors such as secondary infection also remains to be determined.

The variations at *ITGB3*, *CLEC5A* and *CCR5* genes were not associated to severe dengue in our cohort either independently or combined in haplotypes. The protein encoded by *ITGB3* gene (β3 integrin) is abundant in surface receptors of vascular endothelial cells and platelets and has been described as a receptor for DENV in these cells.^18^ *CCR5* plays a role in DENV-2 replication and disease development in human and mouse macrophages.^16^ The *CCR5Δ32* has been previously investigated in our cohort^14^ as well as in a cohort from Australia and, accordingly, no association was found.^30^

*CLEC5A* is critical for dengue-virus-induced lethal disease.^13^ Our group has previously shown an association between a SNP at *CLEC5A* 3’ near-gene (rs1285933) and severe dengue using the same individuals investigated in this study.^14^ The association was replicated later in patients from another Brazilian region.^15^ In the current study, we have increased gene coverage by including additional SNPs as a way to better understand the influence of *CLEC5A* genetic structure in dengue severity. Since no associations were found, the data available to date suggest that the 3’ near gene variation rs1285933 may be the functional variant of this gene.

Results presented here reinforce the association between *MBL2* gene and dengue severity among Brazilians. Our data also highlight the importance of the haplotype context and linkage disequilibrium patterns, since different *MBL2* polymorphisms influence protein expression and activity. Taken together, these results and literature data suggest that *MBL2* levels/activity may influence dengue prognosis.
