# Supplementary material for: Association between MBL2 haplotypes and dengue severity in children from Rio de Janeiro, Brazil
Source: Mem Inst Oswaldo Cruz. 2019 May 23;114:e190004. doi: 10.1590/0074-02760190004 (PMC6534340; doi:10.1590/0074-02760190004)
Supplement: Supplementary file 1 [file 1678-8060-mioc-114-e190004-s.pdf]

TABLE I
Candidate single nucleotide polymorphisms (SNPs) selected for genotyping

Gene	SNP	Chromosome position*	Alleles	Function	EUR**	AFR**
CLEC5A	rs1285950	7:141936763	G > T	Intron region	G: 0.32	G: 0.51
	rs1594777	7:141938010	T > C	Intron region	G: 0.29	G: 0.04
	rs1285948	7:141939670	A > G	Intron region	A: 0.03	A: 0.30
	rs2570407	7:141955092	A > C	Upstream	C: 0.29	C: 0.15
ITGB3	rs4629025	17:47260069	A > T	Intron region	T: 0.64	T: 0.47
	rs884696	17:47263878	A > C	Intron region	A: 0.17	A: 0.27
	rs7209700	17:47273752	A > G	Intron region	G: 0.32	G: 0.40
	rs5918	17:47283364	T > C	Leu59Pro	C: 0.13	C: 0.09
	rs11655943	17:47308274	T > C	Intron region	C: 0.46	C: 0.49
	rs3809865	17:47311220	T > A	3'UTR	T: 0.29	T: 0.26
MBL2	rs2099902	10:52766089	A > G	3'UTR	C: 0.24	C: 0.60
	rs2120131	10:52766258	T > G	3'UTR	G: 0.24	G: 0.45
	rs930509	10:52768593	G > C	Intron region	C: 0.20	C: 0.12
	rs4935047	10:52770307	A > G	Intron region	G: 0.58	G: 0.21
	rs1800451	10:52771466	C > T	Gly57Glu	T: 0.01	T: 0.26
	rs1800450	10:52771475	C > T	Gly54Asp	T: 0.14	T: 0.01
	rs5030737	10:52771482	G > A	Arg52Cys	A: 0.06	A: 0.00
	rs7095891	10:52771701	G > A	Upstream	A: 0.20	A: 0.54
CCR5	rs2856762	3:46371843	C > T	Intron variant	T: 0.09	T: 0.00
	rs3176763	3:46372790	G > T	5'UTR	T: 0.00	T: 0.24
	rs1800940	3:46373082	G > T	Arg60Ser	T: 0.00	T: 0.00
	rs3087253	3:46377198	T > C	Intron variant	C: 0.45	C: 0.16

*: chromosomal position according to GRCh38 genome assembly. **: minor allele frequency from 1000 Genomes Project.

cc BY

TABLE II

Distribution of single nucleotide polymorphisms (SNPs) at *ITGB3*, *CCR5* and *CLEC5A* and analysis of association to dengue severity in children from Rio de Janeiro

Gene	SNP	Genotype/allele	Controls*	Cases*	OR (95%CI; p-value)	OR (95%CI; p-value)**
ITGB3	rs4629025	TT	25 (0.32)	67 (0.38)	Reference	Reference
		TA	40 (0.51)	78 (0.45)	1.23 (0.64 - 2.36; p = 0.53)	1.13 (0.56 - 2.28; p = 0.74)
		AA	13 (0.17)	30 (0.17)	1.24 (0.54 - 2.89; p = 0.61)	1.39 (0.56 - 3.41; p = 0.48)
		Total	78	175		
		Allele T	90 (0.58)	212 (0.61)	Reference	Reference
		Allele A	66 (0.42)	138 (0.39)	1.07 (0.68 - 1.70; p = 0.76)	1.10 (0.67 - 1.82; p = 0.71)
		A carriers	53 (0.68)	108 (0.62)	1.24 (0.67 - 2.27; p = 0.5)	1.19 (0.61 - 2.32; p = 0.61)
	rs884696	CC	50 (0.60)	107 (0.59)	Reference	Reference
		CA	30 (0.36)	62 (0.34)	0.78 (0.42 - 1.45; p = 0.44)	0.80 (0.39 - 1.64; p = 0.54)
		AA	4 (0.05)	13 (0.07)	0.80 (0.24 - 2.69; p = 0.72)	0.93 (0.27 - 3.24; p = 0.91)
		Total	84	182		
		Allele C	130 (0.77)	276 (0.76)	Reference	Reference
		Allele A	38 (0.23)	88 (0.24)	0.87 (0.52 - 1.45; p = 0.59)	0.94 (0.53 - 1.67; p = 0.84)
		A carriers	34 (0.41)	75 (0.41)	0.79 (0.45 - 1.39; p = 0.41)	0.83 (0.43 - 1.59; p = 0.57)
	rs7209700	AA	32 (0.4)	83 (0.48)	Reference	Reference
		AG	40 (0.5)	74 (0.43)	1.32 (0.68 - 2.56; p = 0.41)	1.39 (0.66 - 2.94; p = 0.39)
		GG	8 (0.1)	16 (0.09)	1.21 (0.44 - 3.32; p = 0.71)	1.16 (0.39 - 3.45; p = 0.79)
		Total	80	173		
		Allele A	104 (0.65)	240 (0.69)	Reference	Reference
		Allele G	56 (0.35)	106 (0.31)	1.08 (0.72 - 1.63; p = 0.71)	1.11 (0.67 - 1.83; p = 0.69)
		G carriers	48 (0.6)	90 (0.52)	1.30 (0.69 - 2.42; p = 0.42)	1.33 (0.66 - 2.70; p = 0.42)
	rs5918	TT	69 (0.84)	146 (0.81)	Reference	Reference
		TC	10 (0.12)	30 (0.17)	0.46 (0.18 - 1.16; p = 0.10)	0.35 (0.11 - 1.08; p = 0.07)
		CC	3 (0.04)	4 (0.02)	2.07 (0.41 - 10.43; p =0.38)	2.23 (0.40 - 12.38; p = 0.36)
		Total	82	180		
		Allele T	148 (0.9)	322 (0.89)	Reference	Reference
		Allele C	16 (0.1)	38 (0.11)	0.75 (0.35 - 1.60; p = 0.45)	0.70 (0.30 - 1.65; p = 0.42)
		C carriers	13 (0.16)	34 (0.19)	0.63 (0.28 - 1.39; p = 0.25)	0.55 (0.22 - 1.39; p = 0.21)
	rs11655943	TT	24 (31)	53 (32)	Reference	Reference
		TC	40 (0.51)	68 (41)	1.24 (0.65 - 2.40; p = 0.51)	1.44 (0.72 - 2.88; p = 0.31)
		CC	14 (0.18)	44 (0.27)	0.68 (0.29 - 1.65; p = 0.40)	0.75 (0.29 - 1.91; p = 0.55)
		Total	78	165		
		Allele T	88 (0.56)	174 (0.53)	Reference	Reference
		Allele C	68 (0.44)	156 (0.47)	0.94 (0.59 - 1.50; p = 0.79)	0.99 (0.60 - 1.63; p = 0.96)
		C carriers	54 (0.69)	112 (0.68)	1.67 (0.76 - 3.63; p = 0.20)	1.66 (0.72 - 3.81; p = 0.23)
	rs3809865	AA	42 (0.50)	91 (0.51)	Reference	Reference
		AT	36 (0.43)	70 (0.39)	0.97 (0.52 - 1.81; p = 0.92)	0.74 (0.37 - 1.48; p = 0.40)
		TT	6 (0.07)	18 (0.10)	0.77 (0.27 - 2.14; p = 0.61)	0.65 (0.20 - 2.16; p = 0.48)
		Total	84	179		
		Allele A	120 (0.71)	252 (0.7)	Reference	Reference
		Allele T	48 (0.29)	106 (0.3)	0.95 (0.59 - 1.53; p = 0.83)	0.87 (0.51 - 1.48; p = 0.60)
		T carriers	42 (0.50)	88 (0.49)	0.92 (0.51 - 1.67; p = 0.79)	0.72 (0.37 - 1.41; p = 0.34)

↳

Gene	SNP	Genotype/allele	Controls*	Cases*	OR (95%CI; p-value)	OR (95%CI; p-value)**
CCR5	rs2856762	GG	80 (0.95)	168 (0.91)	Reference	Reference
		GA	4 (0.05)	17 (0.09)	0.47 (0.15 - 1.46; p = 0.19)	0.38 (0.10 - 1.44; p = 0.16)
		AA	0 (0.0)	0 (0.0)	n.d	n.d
		Total	84	185		
		Allele G	164 (0.98)	353 (0.95)	Reference	Reference
		Allele A	4 (0.02)	17 (0.05)	0.55 (0.18 - 1.72; p = 0.30)	0.55 (0.17 - 1.82; p = 0.33)
		A carriers	4 (0.05)	17 (0.09)	0.47 (0.15 - 1.46; p = 0.19)	0.38 (0.10 - 1.44; p = 0.15)
	rs3176763	GG	67 (0.84)	134 (0.77)	Reference	Reference
		GT	13 (0.16)	38 (0.22)	0.79 (0.38 - 1.63; p = 0.52)	1.01 (0.45 - 2.29; p = 0.98)
		TT	0 (0.0)	3 (0.02)	n.d	n.d
		Total	80	175		
		Allele G	147 (0.92)	306 (0.87)	Reference	Reference
		Allele T	13 (0.08)	44 (0.13)	0.81 (0.40 - 1.67; p = 0.57)	1.00 (0.45 - 2.24; p = 0.99)
		T carriers	13 (0.16)	41 (0.24)	0.74 (0.36 - 1.53; p = 0.42)	0.97 (0.43 - 2.19; p = 0.94)
	rs3087253	AA	30 (0.36)	68 (0.38)	reference	Reference
		AG	38 (0.46)	88 (0.50)	0.98 (0.52 - 1.86; p = 0.96)	0.72 (0.35 - 1.47; p = 0.37)
		GG	15 (0.18)	21 (0.12)	1.50 (0.59 - 3.78; p = 0.39)	1.01 (0.35 - 2.96; p = 0.98)
		Total	83	177		
		Allele A	98 (0.59)	224 (0.63)	Reference	Reference
		Allele G	68 (0.41)	130 (0.37)	1.12 (0.71 - 1.76; p = 0.64)	0.97 (0.59 - 1.59; p = 0.90)
		G carriers	53 (0.64)	109 (0.62)	1.07(0.59 - 1.97; p = 0.82)	0.77 (0.39 - 1.52; p = 0.45)
CLEC5A	rs2570407	AA	53 (0.64)	104 (0.59)	Reference	Reference
		AC	27 (0.32)	67 (0.38)	0.84 (0.45 - 1.56; p = 0.58)	0.77 (0.37 - 1.57; p = 0.46)
		CC	3 (0.04)	5 (0.03)	1.74 (0.36 - 8.26; p = 0.49)	1.62 (0.32 - 8.16; p =0.56)
		Total	83	176		
		Allele A	133 (0.80)	275 (0.78)	Reference	Reference
		Allele C	33 (0.20)	77 (0.22)	0.95 (0.57 - 1.61; p = 0.86)	0.94 (0.52 - 1.72; p = 0.20)
		C carriers	4 (0.05)	72 (0.41)	0.89 (0.48 - 1.62; p = 0.69)	0.82 (0.41 - 1.64; p = 0.57)

*: results are shown as N (%). **: odd-ratio (OR) and p-value adjusted for sex and African and Native American genetic ancestries. n.d: not done.

TABLE III

Frequency of *MBL2* haplotypes in the present study and in different populations from 1000 Genomes project

Haplotype number*	Our data (N = 284)**	AFR (N = 661)	AMR (N = 347)	EUR (N = 503)	EAS (N = 504)	SAS (N = 489)
1	0.25	0.19	0.12	0.17	0.11	0.21
2	0.03	0.04	0.02	0.04	0.003	0.007
3	0.06	0.06	0.04	0.05	0.009	0.02
4	0.05	0.09	0.04	0.001	0.07	NA
5	0.12	0.07	0.09	0.18	0.16	0.27
6	0.24	0.12	0.40	0.39	0.45	0.28
7	0.08	0.25	0.02	0.01	NA	0.04
8	0.08	0.01	0.12	0.11	0.14	0.13

Frequencies of each haplotype were determined by maximum likelihood. *: haplotype numbers were defined according to data from the present study, as described in Table III. **: frequencies presented were obtained from the complete sample (N = 87 cases and 197 controls). AFR: African; AMR: Mixed Americans; EUR: European; EAS: East Asians; SAS: South Asians; NA: not available.
